# A Comprehensive, Multi-Scale Dynamical Model of ErbB Receptor Signal Transduction in Human Mammary Epithelial Cells

**DOI:** 10.1371/journal.pone.0061757

**Published:** 2013-04-18

**Authors:** Tomáš Helikar, Naomi Kochi, Bryan Kowal, Manjari Dimri, Mayumi Naramura, Srikumar M. Raja, Vimla Band, Hamid Band, Jim A. Rogers

**Affiliations:** 1 Department of Mathematics, University of Nebraska at Omaha, Omaha, Nebraska, United States of America; 2 College of Information Technology, University of Nebraska at Omaha, Omaha, Nebraska, United States of America; 3 George Washington University School of Medicine, Washington, D. C., United States of America; 4 The Eppley Institute for Research in Cancer and Allied Diseases, Omaha, Nebraska, United States of America; 5 University of Nebraska Medical Center-Eppley Cancer Center, Omaha, Nebraska, United States of America; 6 University of Nebraska Medical Center, Department of Genetics, Cell Biology and Anatomy, College of Medicine, Omaha, Nebraska, United States of America; 7 University of Nebraska Medical Center, Departments of Biochemistry and Molecular Biology; Pathology and Microbiology; and Pharmacology and Experimental Neuroscience, College of Medicine, University of Nebraska Medical Center, Omaha, Nebraska, United States of America; University of Patras, Greece

## Abstract

The non-receptor tyrosine kinase Src and receptor tyrosine kinase epidermal growth factor receptor (EGFR/ErbB1) have been established as collaborators in cellular signaling and their combined dysregulation plays key roles in human cancers, including breast cancer. In part due to the complexity of the biochemical network associated with the regulation of these proteins as well as their cellular functions, the role of Src in EGFR regulation remains unclear. Herein we present a new comprehensive, multi-scale dynamical model of ErbB receptor signal transduction in human mammary epithelial cells. This model, constructed manually from published biochemical literature, consists of 245 nodes representing proteins and their post-translational modifications sites, and over 1,000 biochemical interactions. Using computer simulations of the model, we find it is able to reproduce a number of cellular phenomena. Furthermore, the model predicts that overexpression of Src results in increased endocytosis of EGFR in the absence/low amount of the epidermal growth factor (EGF). Our subsequent laboratory experiments also suggest increased internalization of EGFR upon Src overexpression under EGF-deprived conditions, further supporting this model-generated hypothesis.

## Introduction

EGF receptor (ErbB1) and other members of the ErbB family of receptor tyrosine kinases (RTKs) play essential physiological roles in development and maintenance of epithelial tissues by generating cell proliferation, survival, differentiation and migration signals in response to specific ligands and via the stimulation of several signaling pathways including PI3K/Akt, MAPK, Src, as well as STAT pathways [1], [2]. Activation of ErbB receptors is also linked to the initiation and progression of human cancers. Thus, elucidating signaling pathways that play critical roles in physiological and oncogenic signaling by the ErbB family of receptors is of substantial clinical significance [2]–[5]. Despite substantial progress through experimental studies, in depth mechanistic analyses of the signaling mechanisms of ErbB receptor family have been quite challenging due to the multiple interactions between members of the family, the number of associated effector pathways, and the complexity of regulatory mechanisms [6]. In addition to a multitude of positive signaling pathways triggered by ErbB receptor activation, ErbB receptor signaling is also under regulation by negative feedback mechanisms via receptor endocytosis and recycling/degradation, and this mechanism is critical for normal function [7]. The level of intricacy of the ErbB signaling system is further multiplied by the fact that ErbB signaling pathways are closely intertwined with a number of other signaling pathways such as those downstream of integrins and G-Protein-coupled Receptors [8]. Together, these complexities have hampered our basic understanding of ErbB receptor signaling and our ability to develop treatments for diseases, such as breast cancer, lung cancer, gliomas and others, associated with aberrant ErbB receptor signaling.

An example of the complex biology of ErbB receptor signaling that is highly relevant to their role in oncogenesis involves the non-receptor tyrosine kinase c-Src. The c-Src kinase is overexpressed or hyperactive in a range of human tumors, including breast cancer where as many as 70% cases have been reported with c-Src overexpression along with EGFR/ErbB1 or ErbB2, leading to conjectures of possible synergy between Src and the ErbBs in breast cancer [9]. Indeed, in rodent fibroblasts [9], [10] and more importantly in untransformed human mammary epithelial cells [11] the overexpression of c-Src promotes ErbB1/EGFR-dependent oncogenic transformation. In particular, c-Src is a critical component in the regulation of cell survival, proliferation as well as migration, invasion and metastasis via the regulation of a number of signaling pathways including PI3K/Akt, MAPK, as well as focal adhesion kinase (FAK) [12]. However, the interconnectivity of pathways associated with c-Src and the ErbB signaling has hindered the determination of the mechanisms of ErbB-c-Src synergy in cancer.

These difficulties represent an ideal example of the need for a systems biology approach to ErbB receptor signaling. Because ErbB1/EGFR has been extensively studied over the last several decades, it is perhaps one of the best understood receptor tyrosine kinase systems; this makes it a good candidate for computational modeling [13]. Thus far, several EGFR-based computational models have been created; these have been used in studies focusing on receptor trafficking and endocytosis [14]–[17], ErbB dimerization [18]–[20], as well as the relationships between physiological responses and the receptor activation dynamics [21]–[23]. Several modeling efforts have also been made to better understand the signaling events downstream of EGFR [18], [24]–[28]. In addition, recent efforts also utilized a logical modeling approach to analyze the topology and dynamics of an ErbB signaling network in human liver cells [29], and to identify a potential new drug target, c-MYC, in a model of ErbB receptor-mediated G1/S cell cycle transition [30].

In this work, a new comprehensive, multi-scale logical model of signal transduction in a human mammary epithelial cell (hMEC) is presented. This large-scale dynamical model consists of 245 cellular components and about 1,100 biochemical interactions, and encompasses all ErbB receptor family members, including individual receptor phosphorylation sites, as well as integrin, G-protein-coupled receptor, and stress signaling pathways. The model was constructed manually by collecting and integrating biochemical information from over 800 published papers. One of the main advantages of logical models lies in their scalability; first, they are based on qualitative information available for many cellular components across many cell types and do not depend on kinetic parameters (that are only sparsely available), and second, simulations of logical models are relatively efficient [31], [32], making this approach appropriate for large systems.

Simulations of the model indicate a relatively accurate depiction of the complexities of ErbB signaling by integrating a number of other signaling pathways such as G-protein-coupled receptors (GPCR) and integrins. Following its validation, the model was used to generate predictions about the role of c-Src in EGFR signaling, which were verified experimentally in the laboratory. Finally, the model (including its governing logical expressions as well as all annotations) is available on-line in The Cell Collective software (www.thecellcollective.org; [32]) not only for download in multiple open formats (e.g., SBML; [33]) , but also for live simulations.

## Results

### 1. The hMEC model

**Figure 1:**
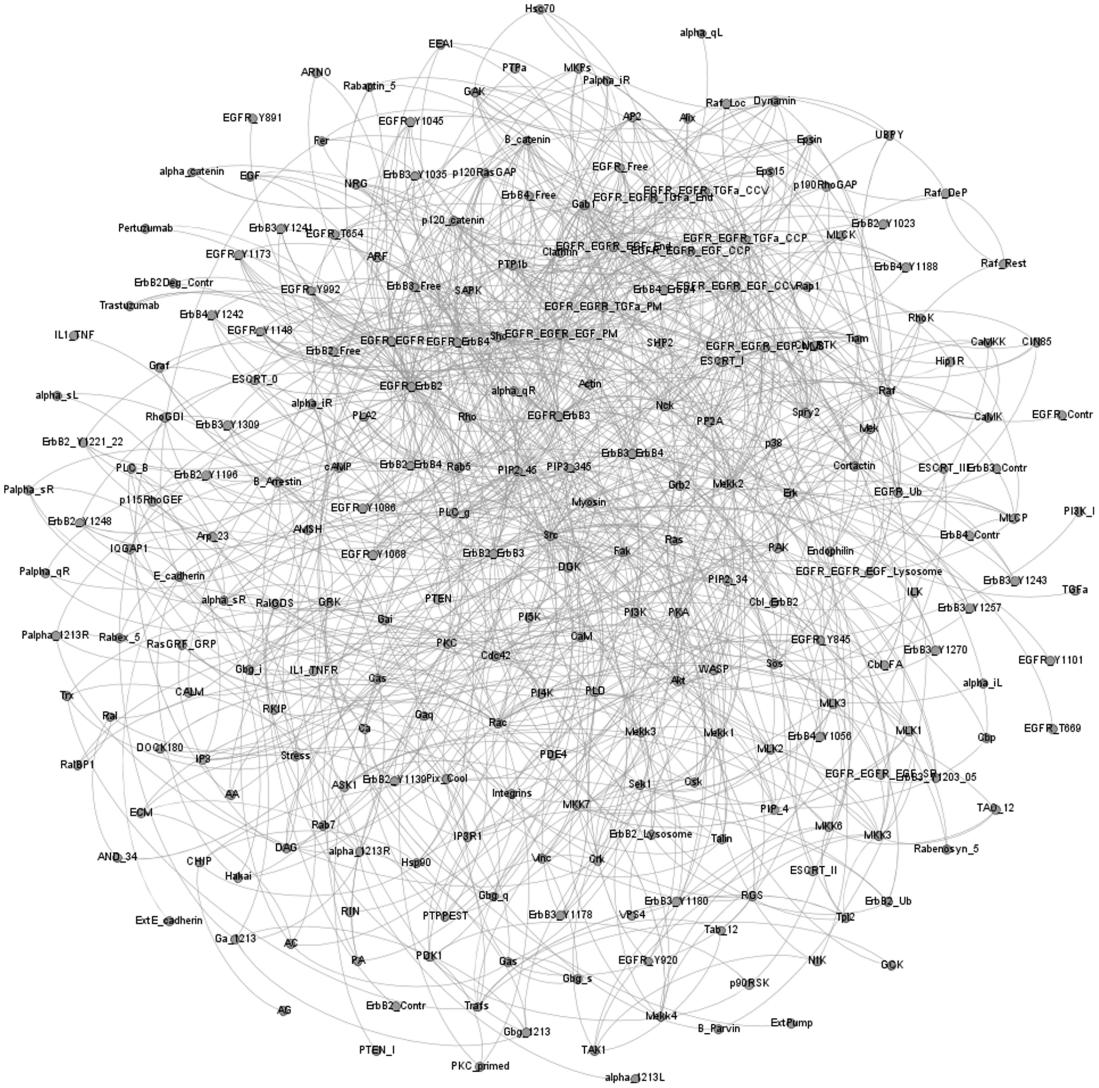
Graph representation of the model. The purpose of this graph is to visualize the complexity of the model, rather than to read the individual interactions. This graph representation of the model was generated in Gephi (www.gephi.org).

The hMEC model for EGFR signaling networks in a human mammary epithelial cell was created by manually collecting information on local biochemical interactions (e.g., protein-protein) from the primary literature (using the same methods as described in [34]). All interactions and logical expressions have been cataloged and annotated, and are available in the Cell Collective software [32]. In addition, all representing logical expressions are available in Supporting Information S1. The model contains a number of integrated signaling pathways at the level of protein-protein as well as -post-translational (namely phosphorylation) site interactions. These pathways include E-cadherin, ErbB (1–4), ErbB1 (EGFR) endocytosis, G-protein-coupled Receptor, integrin, and stress signaling pathways (Figure 1). Detailed description of some of these follows below.

#### ErbB receptors

All known individual ErbB receptors (ErbB1–4), as well as the major ErbB receptor dimers were included in the model. Moreover, the hierarchy of the dimerization process [35] was also captured via the logical functions associated with each receptor node in the model (see the *Model Validation* section). Furthermore, this model goes into an even greater detail; all major (auto) phosphorylation sites of the ErbB receptors were included. This allows for a range of mutational studies at the phosphorylation site level in future studies; for example, this multi-scale property of the mode will enable *in silico* simulations and analyses of system-wide effects of all theoretically possible combinations of virtual knock-out studies of the main phosphorylation sites in a single receptor as well as across all ErbB receptors.

#### EGFR endocytosis

EGFR endocytosis as a potential mechanism of negative regulation through lysosomal degradation is extremely important in relation to oncogenic signaling by ErbB receptors [6]. Modeling receptor endocytosis is not trivial as the same receptor can localize into different areas of the cell (e.g., clathrin-coated (CC) pits, CC vesicles, endosome, etc.), depending on its stage of the endocytic trafficking. The way these multiple localizations are handled using our modeling framework is to represent EGFR with multiple nodes depicting the receptor in the different locations during endocytic traffic. Specifically, the locations included in the model are the following: the plasma membrane, clathrin-coated pits, clathrin-coated vesicles, early endosome, late endosome/multivesicular bodies (MVBs), and the lysosome. The node representing EGFR in the lysosome denotes the degradation process of EGFR. Because results from these models do not attempt to predict exact measurements such as concentrations of ligands used in laboratories, during a simulation, the activity level associated with, for example, the node representing EGFR in the lysosome corresponds to the *degree* of EGFR being degraded in a semi-quantitative fashion. Furthermore, the type of ligand which activates EGFR has an effect on whether the receptor is recycled back to cell surface or degraded upon its internalization. To be able to simulate these effects in addition to the nodes representing the different localizations of the receptor during endocytosis, nodes depicting the receptor being activated by different types of ligands (using EGF or TGFα as prototypes) were added. Thus there is a node that represents EGFR on the plasma membrane activated by EGF and a node for EGFR activated by TGF-alpha. While these differentiations add complexity to the model, they allow the visualization and study of the dynamics of the model in greater detail.

#### Drugs and Antibodies

Therapeutic agents that impact ErbB receptor signaling and/or traffic, such as humanized anti-ErbB2 monoclonal antibodies Herceptin and Pertuzumab, as well as small molecular inhibitors are included in the presented model. These components allow the simulation of mutations known to cause cancer while at the same time introducing a number of different levels and combinations of drugs and observing their effects on the dynamics of the system.

In summary, the initial version of the new model of signal transduction in human MEC comprises 240+ biological species and 1,100+ biochemical interactions. The amount of detail accomplished by including individual phosphorylation sites, various localizations, as well as dimers of ErbB receptors, and the scale in terms of the number of the different pathways included makes this model, to our best knowledge, the most comprehensive dynamic model of signal transduction available to date.

### 2. Model Validation

**Figure 2:**
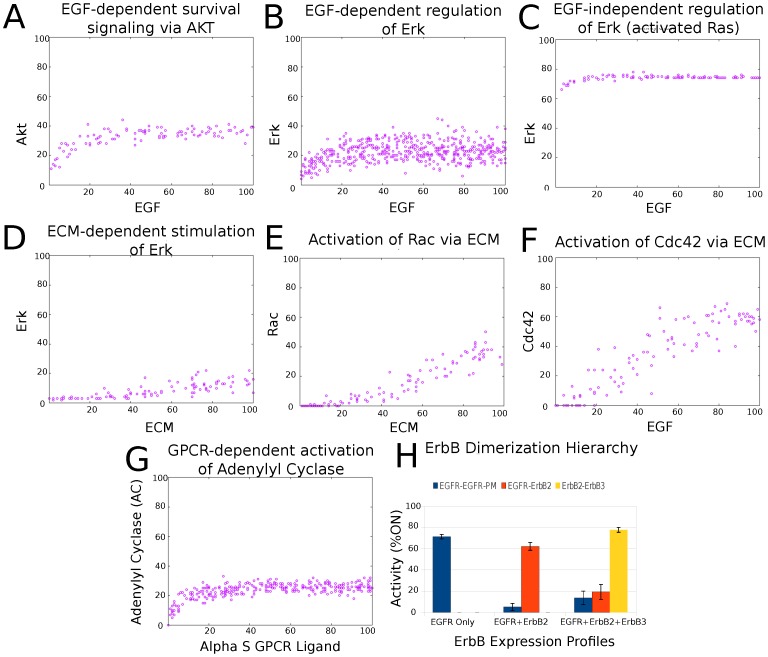
Validation of the MEC model against known cellular phenomena. A) EGF-dependent stimulation of survival signals via activation of Akt. B) Activation of Erk by EGF [52]. Although a positive relationship between Erk and the ligand can be seen, the activation Erk by EGF seems to exhibit a more complex dynamics than the ones seen in the other diagrams (*i.e.*, the positive relationship is exhibited when EGF activity levels are 0–40%). This is, however, not surprising given the complex interactions and cross-communication within the ErbB signaling family. C) Activating mutations of known protooncogenes such as Ras result in growth factor-independent activation of Erk [53]. D) Erk dependency on signaling via integrins by extracellular matrix (ECM) [54]. E and F) Activation of Rac and Cdc42 by ECM [55]. G) Positive relationship between Adenylyl Cyclase (AC) and the G-Protein Coupled Receptor ligand alpha-s [56], [57]. H) Hierarchy of ErbB receptor dimerization. The panel on the left represents a system with EGFR expression alone and, thus, the formation of EGFR homodimers. The expression of ErbB2 (middle panel) results in the shift of the formation (activity) from EGFR homodimers (EGFR-EGFR) to the formation of EGFR-ErbB2 heterodimers. Furthermore, the expression of ErbB 1–3 results in the dominant formation of ErbB2–3 heterodimers [13], [35]. Note that the references refer to classical, qualitative input-output relationships (not necessarily quantitative dose–response curves), and the dose-response curves presented here are intended to demonstrate how the computational model qualitatively reproduces the referenced input-output relationships over a range of stimulus signals.

The model was constructed using only information from the primary literature about *local* interactions. In other words, during the construction phase of the model there was no attempt to determine the local interactions based on any other larger phenotypes or phenomena. However, after the model was completed, verification of the accuracy of the model involved testing it for the ability to reproduce complex input-output phenomena that have been observed in the laboratory. To do this, The Cell Collective's “Dynamical Analysis” simulation feature was used [32]. This simulation component allows users to simulate virtual cells under tens of thousands of cellular conditions, and analyze and visualize the results in terms of input-output dose-response curves that make it easy to determine whether the virtual cell behaves as expected. The presented hMEC model was interrogated to ensure that it is able to reproduce some of the known global biological phenomena as previously observed experimentally (Figure 2), including EGF-induced activation of Akt and Erk, EGF-independent regulation of Erk via activated Ras, integrin-dependent stimulation of Erk, Rac, and Cdc42, G-protein Coupled Receptor activation of adenylyl cyclase, as well as ErbB receptor dimerization hierarchy. Clearly, one cannot expect that *all* phenomena will be replicated by the virtual cell due to the fact that the model does not represent the entire cell. Hence, the reproducibility of certain phenomena by the model only indicates that the model is on the right track.

### 3. Use of the model to form laboratory-testable predictions

**Figure 3:**
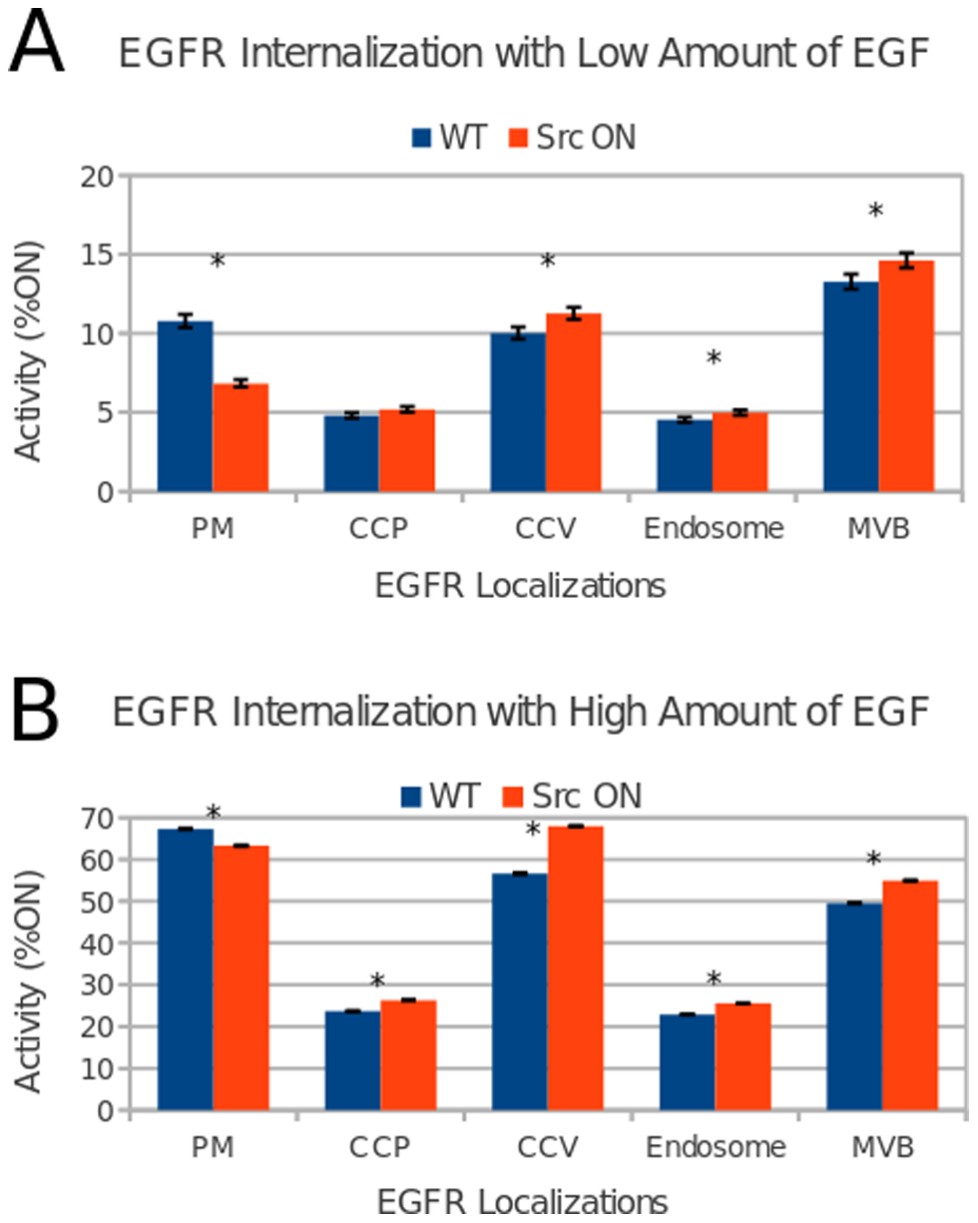
EGFR internalization effects by Src overexpression. Experiments were conducted under conditions for which the expression of ErbB2–4 was turned off and the virtual cell stimulated with EGF. A) Stimulation of the virtual cell with low levels of EGF (0–5%). B) EGF was introduced to the model in randomly selected high activity levels (60–70% ON). * p<0.05 (Student's t-test; *n* = 300; error bars represent the standard error of the mean). Note: PM corresponds to EGFR homodimers on the plasma membrane, CCP represents the homodimer in clathrin-coated pits, CCV – clathrin-coated vesicles, and MVB – EGFR homodimer in late endosome/multivescicular bodies.

Once a functional model of hMEC signaling was completed, we used it to generate predictions about the system that could be subsequently tested in the laboratory. In this work, the conjecture of possible synergy between Src and the ErbBs in breast cancer (described in the introduction) was explored using the model. Specifically, the question examined was whether the internalization of EGFR increases as a result of Src overexpression; this was based on studies done in fibroblasts that showed increased EGFR internalization in response to sub-saturating levels of EGF when Src was overexpressed [36], but this phenomenon has not been investigated in the more relevant MECs nor has it been tested at low levels/in the *absence* of added EGF. To test whether the level of Src activity affects EGFR endocytosis, the node representing c-Src was constitutively activated in the model, using the Cell Collective's Dynamical Analysis feature. The model was then simulated in The Cell Collective, and the activity levels of EGF-activated EGFR homodimer on *i)* the plasma membrane, *ii)* in clathrin-coated pits, *iii)* clathrin-coated vesicles, and *iv)* the endosome were measured. The experiment was first conducted with high levels of EGF to ensure that the model could predict the known increase in internalization of EGFR under that condition. The model was then simulated with low levels of EGF activity (randomly ranging from 0–5%ON). As can be seen in Figure 3, under both high and low levels of EGF, the overexpression of Src (*i.e.*, mutating Src to be constitutively-active) leads to the decrease of the activity levels of the node representing EGFR homodimers on the plasma membrane, while increasing the % ON levels of nodes representing the receptor during the internalization process. Thus the model indicates that Src overexpression will lead to increased internalization of EGFR even in the absence (or low levels) of EGF, a result that we assessed and confirmed experimentally as discussed in the next section.

### 4. Verification of model-generated predictions

**Figure 4:**
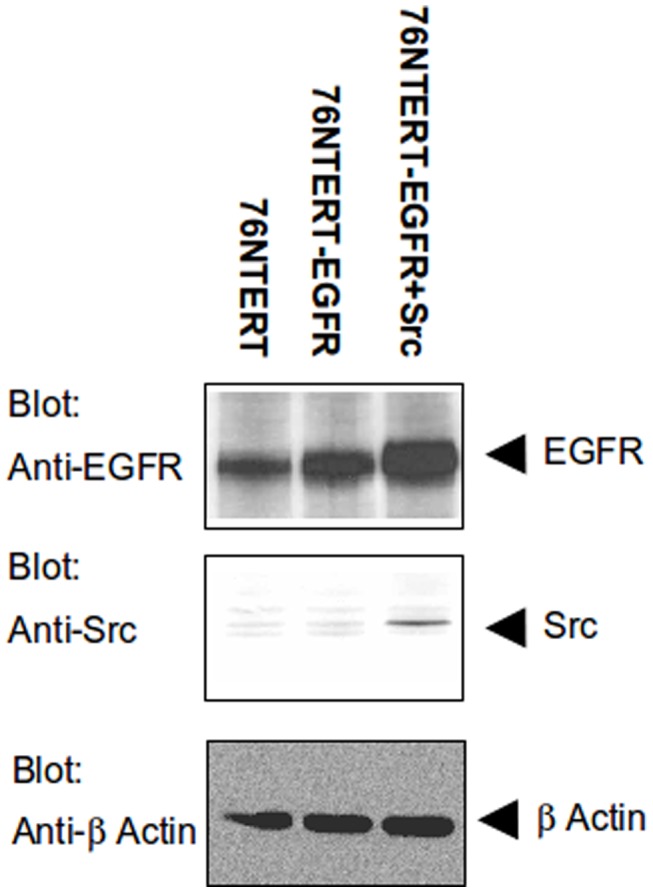
Src overexpression does not affect total EGFR levels. Parental 76N-TERT cells or its EGFR or EGFR + Src transductants were EGF deprived by culture in the EGF-deficient D3 medium for 48 hrs and cell lysates were prepared. 50 µg aliquots of cell lysate protein were run on an 8% SDS PAGE gel and immunoblotted with anti-EGFR (top panel) or anti-Src (middle panel). Membrane was re-probed with anti-beta actin (bottom panel) to ensure equal loading.

**Figure 5:**
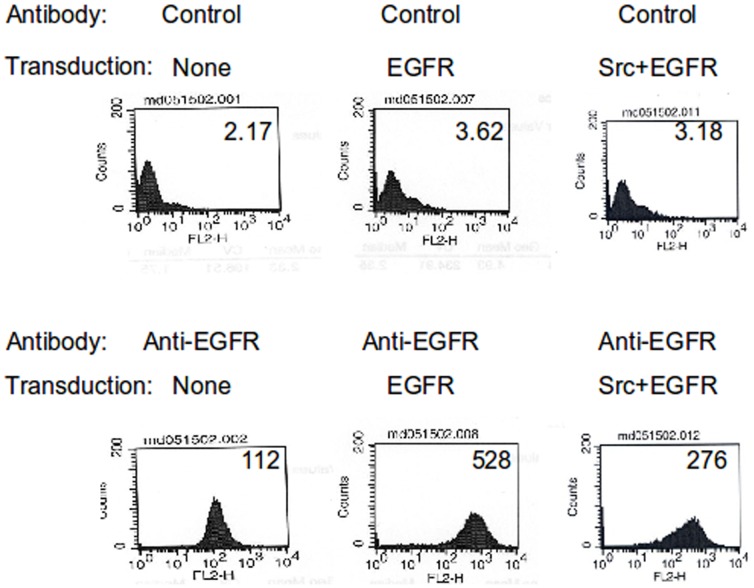
Surface expression of EGFR is reduced in Src-transfected cells. FACS analysis of the cell surface expression of EGFR in MEC transductants shows (in row 2) that Src overexpression leads to a reduction in the level of EGFR at the cell surface despite higher total EGFR levels detected biochemically (shown in Figure 3). Cells were EGF-deprived by culture in D3 medium for 48 h, after which single cell suspensions were prepared with trypsin/EDTA. Live cells were stained with isotype-matched control monoclonal antibodies or with anti-EGFR monoclonal antibody for 1 hr.., washed and incubated with the secondary antibody (PE-conjugated) for 45 min., and analyzed by FACS to determine cell surface levels of EGFR. Numbers in the right top corner of each FACS panel indicate median fluorescence channel intensity (higher numbers indicating higher levels and vice versa). Initial experiments (not shown) established the specificity of antibodies.

We sought to verify whether the predicted enhancement of the intracellular (endocytosed) EGFR pools is seen in an actual human MEC model in laboratory experiments. The overall levels of ectopically expressed EGFR and Src in 76NTERT human MEC lines retrovirally transuced with EGFR or EGFR plus Src (using retroviral infection) [37] were assessed using Western blotting. As is clear in Figure 4, increased Src and EGFR levels are observed in the appropriate transductants. As expected, both the EGFR alone transduced as well as the EGFR plus Src transduced cell lines showed higher EGFR levels compared to the parent 76NTERT cell line (Figure 4; results further explained in the caption). Notably, the total levels of biochemically detected EGFR in the EGFR plus Src transductant (lane 3) exceed the levels of EGFR detectable in the EGFR alone transductant. In contrast, Fluorescence-activated cell sorting (FACS) based analysis that was designed to selectively measure the levels of EGFR on the cell surface (FACS analysis was done on live cells without permabilizing them; under these conditions, the staining antibody is excluded from endocytosed pools of EGFR) demonstrated that, in the absence of added EGF, there is dramatically reduced expression of EGFR on the surface of MECs overexpressing Src (Figure 5, third panel of the second row; indicated by the lower median fluorescence channel values along the X-axis – MFI = 276) compared to EGFR-only transduced MEC controls (Figure 5, second panel of the second row, which expectedly shows a markedly higher median channel value of 528 compared to112 in untransduced control in the first panel). Thus, the prediction from the hMEC model, that higher levels of Src introduced into MECs will lead to a lower proportion of EGFR at the cell surface even in the absence of EGF, *i.e.*, EGFR is internalized, is fully verified by experimental analyses of an actual mammary epithelial cell system.

## Discussion

The critical roles of ErbB receptors in physiological processes and the importance of their overexpression and/or hyperactivity in the initiation and progression of cancers makes analyses of these receptor tyrosine kinases extremely significant. EGFR is also the most highly studied prototype of receptor tyrosine kinase signaling. Therefore, predictive computational modeling of signaling downstream of these receptors in the context of positive and negative loops and cross-talk with other receptor systems are likely to greatly enhance our fundamental knowledge of cellular signaling in health and disease. These models can also provide a more informed platform for therapeutic targeting of aberrant ErbB signaling in cancer to reduce treatment failures and to stem the emergence of resistance. Here, we present a new and most comprehensive computational model of ErbB receptor signaling in a human mammary epithelial cell, use simulations to make a specific prediction on the biological behavior of a hMEC under experimental conditions that model the overexpression of EGFR and Src as seen in human cancers, and then use laboratory experiments to fully verify the prediction.

It is well-established that EGFR and c-Src tyrosine kinase are co-overexpressed in human cancers, and experimental modeling of their co-overexpression in untransformed hMECs in the laboratory leads to their collaborative promotion of oncogenesis [11]. Studies in such systems will be greatly enhanced by generating hypotheses on the interactions of the two oncogenic proteins that can be experimentally tested. As RTK signaling is spatio-temporally regulated in part by the subcellular location of activated receptors in different endocytic compartments which serves as a critical determinant of the level and diversity of their signaling outputs [37], [38]. Therefore, we used the computational model that we have developed to specifically address questions of endocytic localization of EGFR under the influence of Src.

It is remarkable that the model could make an accurate *de novo* prediction that the elevated Src activity will promote the localization of EGFR in internal compartments of the endocytic pathways even in the absence of added ligand. We used a cell system in which we utilized an externally-introduced EGFR (under a viral promoter) to test this prediction. Even though the total levels of EGFR are substantially higher in the cell line that co-overexpresses Src (Figure 4), the levels of EGFR on the cell surface are dramatically reduced (Figure 5); thus, a vast majority of EGFR in these cells is predicted to be in endocytic compartments even without an added ligand. Under normal conditions, ligands such as EGF cause rapid internalization of EGFR and its rapid degradation in the lysosome while other ligands such as TGFalpha or amphiregulin promote less degradation with more recycling [39]. Notably, increased internal pools of EGFR are a feature of oncogenic mutants of EGFR; we have recently shown that human non-small cell lung cancer cell lines harboring EGFR mutants with activating kinase domain mutations show increased levels of active EGFR within intracellular endocytic compartments when cultured without external EGF [40]. Remarkably, the internalized pool of oncogenic EGFR co-localized with active Src [40] and the interaction of mutant EGFR and Src was required for efficient oncogenic transformation of fibroblasts [41]. Thus, our current model-based prediction followed by experimental verification in a relevant cell model in the laboratory should open a productive lead to further experimentation towards understanding how Src and EGFR cooperate in oncogenesis. Importantly, the verified experimental findings can then be incorporated back into our computational model to enhance its ability to make further predictions and efforts along these lines are underway.

The use of computational models to generate experimentally testable hypotheses in an information flow cycle from laboratories to computational models and back to laboratories [42] is an important dynamic that will define the future of computational systems biology. However, the sheer size and complexity of biochemical and biological processes poses a barrier for one person or group to create and/or expand in an effective way large-scale dynamical models of these systems. While the presented model is one of the largest computational models created, it merely represents a small fraction of the cell. Similar to Wikipedia and open source software – both of which are centered around large amounts of knowledge that could not have originated from one single person or group, one way to create larger computational models of biological process, ones that have the potential to eventually lead to whole-cell models, is to engage the scientific community in a collaborative fashion. Hence, the presented hMEC model has been made available in The Cell Collective software which was designed precisely to enable such a collaborative and collective approach to systems biology [32]. The model is available to the entire scientific community via the software for further expansion, refinements, as well as simulations and analyses. The user-friendly interface of the software allows users to make changes to the model without any need to enter complex mathematical equations or computer code, making it accessible to experimental scientists who have the most intimate knowledge of the local data to improve and grow this model (and others available in the software platform; e.g., [30], [43], [44]).

## Materials and Methods

### 1. Model construction via The Cell Collective

The presented model is based on a common qualitative (discrete) modeling technique where the regulatory mechanism of each node is described by a Boolean expression (for more comprehensive information on Boolean modeling see for example [45], [46]). The construction of the model was accomplished using The Cell Collective (www.thecellcollective.org; [32]), a collaborative modeling platform for large-scale biological systems. The platform allows users to construct and simulate large-scale computational models of various biological processes based on qualitative interaction information using the platform's Bio-Logic Builder which converts the entered qualitative biochemical information into Boolean expressions in the background [47]. (Though the Boolean expressions for any model created in the platform can be downloaded from the website.) This non-technical creation and representation of the individual interactions in the model make it especially easy for experimental biologists to contribute to the creation of the model without the need for training in the underlying mathematical formalisms. The model has been exported and is also available as part of this manuscript in a SBML format for qualitative models (Supporting Information S2).

The Cell Collective's Knowledge Base component was also used to catalog and annotate all biochemical/biological information for signaling in hMECs as mined from the primary literature. Each model species in the Knowledge Base has its own wiki-like page where information on the individual interactions is stored, including references.

### 2. Model Simulations and Analysis

In addition to the model building and cataloging process, The Cell Collective platform was also used to perform all computational simulations of the hMEC model. While the dynamical model is based on a discrete (*i.e.*, Boolean) formalism, as can be seen in the results, the simulation input and output data are continuous. This was accomplished by converting the digital output of the model simulations to % activity (% ON) which ranges (for each model component) from 0 to 100 [31], [34]. It is important to note that the % ON doesn't directly correspond to the biological concentration or any other measurable property, rather the % ON provides a semi-quantitative measure to describe the relative activity level of a particular protein. As such, the model output (species activity levels) is compared to previously published experimental findings as well as the experimental results presented herein by assessing the directionality of the changes (up-/down-regulation) of species activity relative to the wild-type. All simulations were conducted using a biologically relevant initial condition as discussed in [34]; this condition is also accessible via The Cell Collective software. All *in silico* experiments were performed under external conditions (Table 1) that were optimized for the particular experiment [34].

**Table 1:** External simulation conditions.

Stimulus/Simulation Experiment	Fig. 2A	Fig. 2B	Fig. 2C	Fig. 2D	Fig. 2E	Fig. 2F	Fig. 2G	Fig. 2H	Fig. 3A	Fig. 3B
**alpha_1213L**	40–60	72	72	60–70	5–65	70–80	36	26	20–30	20–30
**alpha_iL**	0–10	11	11	70–80	30–80	40–50	69	97	90	100
**alpha_qL**	0–10	37	37	10–20	50–100	90–100	77	4	1	5
**alpha_sL**	40–60	48	48	10–20	55–95	50–60	0–100	55	50	60
**ECM**	0–100	88	88	0–100	0–100	0–100	99	94	90	100
**EGF**	0–100	0–100	0–100	0	0–100	1–10	10	90–100	0–5	60–70
**EGFR_Contr**	100	100	100	100	100	100	100	90–100	100	100
**ErbB2_Contr**	0	0	0	0	0	0	0	0/90–100/90–100	0	0
**ErbB2Deg_Contr**	0	0	0	0	0	0	0	0/25–35/25–35	0	0
**ErbB3_Contr**	0	0	0	0	0	0	0	0/0/90–100	0	0
**ExtPump**	40–60	12	12	1–10	80–100	90–100	94	76	70–80	70–80
**IL1_TNF**	2	2	2	2	2	2	2	2	2	2
**Stress**	2	2	2	2	2	2	2	2	2	2

All values represent (%ON) activity levels. Stimuli not used in these experiments were set to 0 and are not listed in the table. Note that while some external conditions consist of ranges and others of specific values, we find that most experiments are not sensitive to specific values. Using the values provided in this table, all simulated experiments of the model can be reproduced in The Cell Collective software.

### 3. Establishment of mammary epithelial cell lines overexpressing EGFR or EGFR plus Src

Human telomerase reverse transcriptase (TERT) immortalized 76N normal human MEC line 76N-TERT has been previously described [48], [49]. These cells were cultured in DFCI-1 medium and the human EGFR or EGFR plus c-Src were overexpressed in these cells using retroviral infection as described previously [50].

### 4. Assessment of Src overexpression and EGFR levels using Western blotting

Parental 76NTERT cell line or its EGFR or EGFR + Src transductants were growth factor deprived for 48 h (with medium change each day) in EGF-deficient D3 medium (DFCI-1 medium lacking insulin, hydrocortisone, EGF and bovine pituitary extract) [51], Cell lysates were prepared in a Triton X-100-based lysis buffer, and 50 µg aliquots of cell lysate protein were run on an 8% SDS PAGE gel and immunoblotted with anti-EGFR, anti-Src and anti-beta actin antibodies, as described [50].

### 5. Assessment of surface expression of EGFR

Cells were cultured under EGF-deprivation conditions as above for 48 h, and single cell suspensions were prepared by releasing cells from tissue culture plates with trypsin/EDTA. Live cells were stained on ice with isotype-matched control monoclonal antibodies or with anti-EGFR monoclonal antibody (clone 528; ATCC) for 1 h, washed and incubated with the secondary antibody (PE-conjugated anti-mouse IgG) for 45 min followed by FACS analysis (using a FACSCalibur instrument) to determine the relative cell surface EGFR levels.

## Supporting Information

Supporting Information S1 A comprehensive, multi-scale dynamical model of ErbB receptor signal transduction in human mammary epithelial cells
(DOC)

Supporting Information S2 SBML model representation of signal transduction epithelial cells
(SBML)
