# Supplementary material for: A Comprehensive, Multi-Scale Dynamical Model of ErbB Receptor Signal Transduction in Human Mammary Epithelial Cells
Source: PLoS One. 2013 Apr 18;8(4):e61757. doi: 10.1371/journal.pone.0061757 (PMC3630219; doi:10.1371/journal.pone.0061757)
Supplement: Supporting Information S1 — A comprehensive, multi-scale dynamical model of ErbB receptor signal transduction in human mammary epithelial cells (DOC) [file pone.0061757.s001.doc]

**Supporting Information S1: A comprehensive, multi-scale dynamical model of ErbB receptor signal transduction in human mammary epithelial cells**

Tomáš Helikar^(1)^, Naomi Kochi^(1)^, Bryan Kowal^(2)^, Manjari Dimri^(3)^, Mayumi Naramura^(4,5,6)^, Srikumar M Raja^(4,5)^, Vimla Band^(4,5,6)^, Hamid Band^(4-7)^, Jim A Rogers^(1,6,*)^

^1^ Department of Mathematics, University of Nebraska at Omaha, Omaha, NE, USA

^2^ College of Information Technology, University of Nebraska at Omaha, Omaha, NE, USA

^3^ George Washington University School of Medicine, Washington, DC, USA

^4^ The Eppley Institute for Research in Cancer and Allied Diseases, Omaha, NE, USA

^5^ University of Nebraska Medical Center-Eppley Cancer Center, Omaha, NE, USA

^6^ University of Nebraska Medical Center, Department of Genetics, Cell Biology and Anatomy, College of Medicine, Omaha, NE, USA

^7^ University of Nebraska Medical Center , Departments of Biochemistry and Molecular Biology; Pathology and Microbiology**;** and Pharmacology and Experimental Neuroscience, College of Medicine, University of Nebraska Medical Center, Omaha, NE, USA

* Corresponding Author

| **Species** | **Logic Expression** |
| --- | --- |
| EGFR_EGFR_TGFa_CCV | (EGFR_EGFR_TGFa_CCP & ((Clathrin | GAK) & (Actin & Dynamin) & (AP2 & PIP2_45))) | (EGFR_EGFR_TGFa_CCV & !EGFR_EGFR_TGFa_End) |
| Erk | Mek | ((Erk & !PP2A) & !MKPs) |
| Src | ((Cas & PTP1b) & !(Csk & Src)) | ((alpha_sR & B_Arrestin) & !(Csk & Src)) | ((Gai & B_Arrestin) & !(Csk & Src)) | ((Fak & PTP1b) & !(Csk & Src)) | ((PTPa & !Src) & !(Csk & Src)) | ((EGFR_Y1148) & !(Csk & Src)) | ((EGFR_Y992) & !(Csk & Src)) | (EGFR_Y1086 & !(Csk & Src)) | ((Gas & B_Arrestin) & !(Csk & Src)) |
| MLK2 | (Rac & SAPK) | (Cdc42 & SAPK) |
| EGFR_EGFR_EGF_Lysosome | (ESCRT_III & (Alix & Rab7 & (Eps15 | VPS4))) | (EGFR_EGFR_EGF_MVB & (Alix & Rab7 & (Eps15 | VPS4))) |
| ErbB3_Y1241 | (ErbB2_ErbB3) | (ErbB3_ErbB4) | (EGFR_ErbB3) |
| RalGDS | (((alpha_qR & B_Arrestin) & !(Ras & (PDK1 & PIP3_345))) & !PKC) | (((alpha_1213R & B_Arrestin) & !(Ras & (PDK1 & PIP3_345))) & !PKC) | (((alpha_sR & B_Arrestin) & !(Ras & (PDK1 & PIP3_345))) & !PKC) | (((alpha_iR & B_Arrestin) & !(Ras & (PDK1 & PIP3_345))) & !PKC) |
| EGFR_Y1148 | EGFR_EGFR_TGFa_PM | EGFR_EGFR_EGF_PM | EGFR_ErbB4 | EGFR_ErbB2 | EGFR_ErbB3 | EGFR_EGFR |
| PLC_B | Gaq | ((Gbg_i & PLC_B) & !(PKA & !Gaq)) |
| B_Arrestin | Palpha_sR | Palpha_1213R | Palpha_iR | Palpha_qR |
| AA | PLA2 |
| Rap1 | (CaMK & ((!Gai | !Rap1) & Src & cAMP)) | (PKA & (!Gai | !Rap1) & (Src & cAMP) |
| ErbB4_Free | ErbB4_Contr | ((((ErbB4_Free & !ErbB4_ErbB4) & !ErbB2_ErbB4) & !ErbB3_ErbB4) & !EGFR_ErbB4) |
| EGFR_Y1086 | EGFR_EGFR_TGFa_PM | EGFR_EGFR_EGF_PM | EGFR_ErbB4 | EGFR_ErbB2 | EGFR_ErbB3 | EGFR_EGFR |
| Mekk1 | (Rho & (Grb2 | Shc)) | Ras | GCK | Rac | (NIK & (Grb2 | Shc)) | Cdc42 | (Grb2 & Shc) | Trafs |
| EGFR_Free | (EGFR_Free & !(EGFR_Free & (EGFR_EGFR | EGFR_EGFR_EGF_PM | EGFR_EGFR_TGFa_PM | EGFR_ErbB2 | EGFR_ErbB3))) | (EGFR_EGFR_TGFa_End & !(EGFR_Free & (EGFR_EGFR | EGFR_EGFR_EGF_PM | EGFR_EGFR_TGFa_PM | EGFR_ErbB2 | EGFR_ErbB3))) | (EGFR_Contr & !(EGFR_Free & (EGFR_EGFR | EGFR_EGFR_EGF_PM | EGFR_EGFR_TGFa_PM | EGFR_ErbB2 | EGFR_ErbB3))) |
| PLC_g | AA | (EGFR_Y1173 & (EGFR_EGFR | EGFR_EGFR_EGF_CCP | EGFR_EGFR_EGF_CCV | EGFR_EGFR_EGF_End | EGFR_EGFR_EGF_MVB | EGFR_EGFR_EGF_PM | EGFR_EGFR_TGFa_CCP | EGFR_EGFR_TGFa_CCV | EGFR_EGFR_TGFa_End | EGFR_EGFR_TGFa_PM | EGFR_ErbB2 | EGFR_ErbB3 | EGFR_ErbB4)) | (EGFR_Y992 & (EGFR_EGFR | EGFR_EGFR_EGF_CCP | EGFR_EGFR_EGF_CCV | EGFR_EGFR_EGF_End | EGFR_EGFR_EGF_MVB | EGFR_EGFR_EGF_PM | EGFR_EGFR_TGFa_CCP | EGFR_EGFR_TGFa_CCV | EGFR_EGFR_TGFa_End | EGFR_EGFR_TGFa_PM | EGFR_ErbB2 | EGFR_ErbB3 | EGFR_ErbB4)) | (EGFR_Y1068 & (EGFR_EGFR | EGFR_EGFR_EGF_CCP | EGFR_EGFR_EGF_CCV | EGFR_EGFR_EGF_End | EGFR_EGFR_EGF_MVB | EGFR_EGFR_EGF_PM | EGFR_EGFR_TGFa_CCP | EGFR_EGFR_TGFa_CCV | EGFR_EGFR_TGFa_End | EGFR_EGFR_TGFa_PM | EGFR_ErbB2 | EGFR_ErbB3 | EGFR_ErbB4)) |
| ARF | (ARF & !PIP2_45) | (PIP3_345 & !PIP2_45) | (PIP2_45 & ((ARNO | PIP3_345) & !ARF)) | (ARNO & !PIP2_45) |
| SHP2 | Gab1 |
| Talin | (Talin & !Src) | (PIP2_45 & !Talin) |
| ErbB4_ErbB4 | NRG & ErbB4_Free & !ErbB2_Free |
| cAMP | AC & !PDE4 | cAMP & !PDE4 |
| ErbB3_Y1178 | ErbB2_ErbB3 | ErbB3_ErbB4 | EGFR_ErbB3 |
| PIP2_45 | PIP2_45 & !(PI3K & PIP2_45) | PTEN & PIP3_345 | PI4K & PI5K |
| Crk | ((Cas & (Fak | Src)) & !PTPPEST) |
| Clathrin | CALM & PIP2_45 | ESCRT_0 | Src | Hip1R | AP2 | Epsin & PIP2_45| GAK |
| Endophilin | CIN85 | Endophilin & PIP2_45 | Epsin | Eps15 |
| MLCK | (((CaM & (!PAK & !PKA)) & !PKA) & !PAK) | (((Erk & (!PAK & !PKA)) & !PKA) & !PAK) |
| EGFR_ErbB2 | (Pertuzumab & EGFR_ErbB2) | ((EGF & (!EGFR_ErbB2 & !EGFR_ErbB2 & !EGFR_T654 & (!ErbB3_Free | !NRG) & EGFR_Free & ErbB2_Free)) & !Pertuzumab) | ((EGFR_ErbB2 & !Trastuzumab) & !(ErbB2Deg_Contr & EGFR_ErbB2)) |
| MLK1 | Rac | Cdc42 |
| Fak | (Integrins & Talin & !(PTEN & Fak)) | (Src & Fak & !(PTEN & Fak)) |
| MKPs | (p38 & cAMP) | (SAPK & cAMP) | (Erk & cAMP) |
| Mekk4 | Rac | Cdc42 |
| Gbg_i | alpha_iR & !Gai & !Gbg_i | Gai |
| ErbB3_Y1035 | ErbB2_ErbB3 | ErbB3_ErbB4 | EGFR_ErbB3 |
| CaMK | CaMKK & CaM |
| PI4K | Rho | Gaq | ARF | Gai | PKC |
| Alix | ESCRT_III | ESCRT_I |
| Hip1R | CIN85 | Clathrin |
| Raf_DeP | (PP2A & (!Raf_DeP & Raf_Rest)) | (Raf_DeP & !Raf_Loc) |
| UBPY | ESCRT_III | Alix | ESCRT_I |
| ASK1 | Trx |
| Mek | (Tpl2 & !(PP2A & Mek)) | ((Mekk3 & Raf) & !(PP2A & Mek)) | ((Raf & Tpl2) & !(PP2A & Mek)) | ((Mekk2 & Raf) & !(PP2A & Mek)) | ((Mekk1 & Raf) & !(PP2A & Mek)) | (PAK & Tpl2& !(PP2A & Mek)) |
| MKK7 | Mekk4 & ASK1 | Mekk3 & ASK1 | Mekk2 & ASK1 | Mekk1 & ASK1 | MLK1 & ASK1 | MLK2 & ASK1 | MLK3 & ASK1 |
| RalBP1 | Ral |
| Gaq | Gaq & !PLC_B & !RGS & Gbg_q | alpha_qR & !Gaq & !Gbg_q |
| Vinc | Talin & Src | Actin & !PIP2_45 & Talin & Vinc |
| EGFR_EGFR_EGF_PM | EGFR_Free & !EGFR_T654 & EGF & !ErbB2_Free & !EGFR_EGFR_EGF_CCP | EGFR_EGFR_EGF_PM & !EGFR_EGFR_EGF_CCP) |
| Pix_Cool | PIP2_34 & B_Parvin | PIP3_345 & B_Parvin |
| EGFR_ErbB3 | EGF & !EGFR_T654 & !ErbB2_Free & EGFR_Free & ErbB3_Free & NRG |
| EGFR_EGFR_EGF_MVB | EGFR_EGFR_EGF_End | EGFR_EGFR_EGF_MVB & !EGFR_EGFR_EGF_Lysosome |
| Gab1 | ((Gab1 & (PIP3_345 & (EGFR_EGFR | EGFR_EGFR_EGF_CCP | EGFR_EGFR_EGF_CCV | EGFR_EGFR_EGF_End | EGFR_EGFR_EGF_MVB | EGFR_EGFR_EGF_PM | EGFR_EGFR_TGFa_CCP | EGFR_EGFR_TGFa_CCV | EGFR_EGFR_TGFa_End | EGFR_EGFR_TGFa_PM | EGFR_ErbB2 | EGFR_ErbB3 | EGFR_ErbB4))) & !SHP2) | ((Grb2 & (!Gab1 & ((EGFR_EGFR | EGFR_EGFR_EGF_CCP | EGFR_EGFR_EGF_CCV | EGFR_EGFR_EGF_End | EGFR_EGFR_EGF_MVB | EGFR_EGFR_EGF_PM | EGFR_EGFR_TGFa_CCP | EGFR_EGFR_TGFa_CCV | EGFR_EGFR_TGFa_End | EGFR_EGFR_TGFa_PM | EGFR_ErbB2 | EGFR_ErbB3 | EGFR_ErbB4)))) & !SHP2) |
| ErbB2_ErbB3 | (ErbB2_ErbB3& !(ErbB2_Lysosome & ErbB2_ErbB3)) | (NRG & ErbB2_Free & ErbB3_Free & !Pertuzumab & !(ErbB2_Lysosome & ErbB2_ErbB3)) |
| PDK1 | Src | p90RSK |
| RasGRF_GRP | (DAG & Cdc42) | (CaM & Cdc42) |
| EGFR_Y992 | (EGFR_EGFR_TGFa_PM & !(SHP2 & EGFR_Y992)) | (EGFR_EGFR_EGF_PM & !(SHP2 & EGFR_Y992)) | (EGFR_ErbB4 & !(SHP2 & EGFR_Y992)) | (EGFR_ErbB2 & !(SHP2 & EGFR_Y992)) | (EGFR_Y992 & !(SHP2 & EGFR_Y992)) | (EGFR_ErbB3 & !(SHP2 & EGFR_Y992)) | (EGFR_EGFR & !(SHP2 & EGFR_Y992)) |
| TAO_12 | Stress |
| Gas | (alpha_sR & !Gas & !Gbg_s & !PKA) | (Gbg_s & !RGS & Gas) |
| EGFR_EGFR_EGF_CCV | ((EGFR_EGFR_EGF_CCV) & !(EGFR_EGFR_EGF_End)) | (EGFR_EGFR_EGF_CCP & Actin & Dynamin & !EGFR_EGFR_EGF_End) |
| p90RSK | Erk & !p90RSK & PDK1 |
| Rabaptin_5 | Rab5 |
| PAK | (((Src & PAK & (Cdc42 | Rac)) & !PTP1b) & !PKA) | ((Rac & (Grb2 | (Nck & !Akt))) & !PKA) | ((Cdc42 & (Grb2 | (Nck & !Akt))) & !PKA) |
| AC | Integrins & ECM & Gas & Gbg_i |
| Cbl_ErbB2 | Trastuzumab & (EGFR_ErbB2 | ErbB2_ErbB3 | ErbB2_ErbB4) |
| ErbB4_Y1056 | ErbB4_ErbB4 | ErbB2_ErbB4 | ErbB3_ErbB4 | EGFR_ErbB4 |
| EGFR_Y920 | Src & EGFR_EGFR_EGF_PM |
| AND_34 | Cas |
| Rac | ((Rac & !RalBP1) & !(RalBP1 & Rac)) | ((DOCK180 & ECM & Integrins) & !(RalBP1 & Rac)) | (Pix_Cool & ((!Gbg_i & (!Rac & Cdc42 & ECM & Integrins)) | ((!PAK) & (((!DOCK180 & !Rac & !Rac & !RasGRF_GRP & !Tiam)) & Cdc42 & ECM & Integrins)) | ((Gbg_i & PAK) & (!Rac & ECM & Integrins)))) | ((Tiam & ECM & Integrins) & !(RalBP1 & Rac)) | ((RasGRF_GRP & ECM & Integrins) & !(RalBP1 & Rac)) |
| alpha_1213R | ((Palpha_1213R & !B_Arrestin) & !(B_Arrestin & ((!Palpha_1213R & !alpha_1213L & !alpha_1213R) | Palpha_1213R))) | (alpha_1213R & !(B_Arrestin & (((!Palpha_1213R & !alpha_1213L & !alpha_1213R)) | Palpha_1213R))) | (alpha_1213L & !(B_Arrestin & ((!Palpha_1213R & !alpha_1213L & !alpha_1213R) | Palpha_1213R))) |
| CaMKK | CaM |
| alpha_catenin | B_catenin |
| MLK3 | IL1_TNFR | Rac | Cdc42 |
| EGFR_Y1173 | EGFR_EGFR_TGFa_PM | EGFR_EGFR_EGF_PM | EGFR_ErbB4 | EGFR_ErbB2 | EGFR_ErbB3 | EGFR_EGFR |
| p120RasGAP | ((PIP2_34 & !Src) & !Fak) | ((PIP3_345 & !Src) & !Fak) | ((PIP2_45 & !Src) & !Fak) | (EGFR_Y992 & !Src & !Fak) |
| ErbB3_Y1243 | ErbB2_ErbB3 | ErbB3_ErbB4 | EGFR_ErbB3 |
| TAK1 | Tab_12 |
| Cdc42 | ((Cdc42 & ((!Graf & !RalBP1 & !p190RhoGAP) & IQGAP1)) & !(RhoGDI & Src)) | ((Pix_Cool & (Cdc42 | Gbg_i & PAK & !Rac)) & !(RhoGDI & Src)) |
| p190RhoGAP | (Src & (!p120RasGAP | !p190RhoGAP | Fak)) | (Fak & Src) |
| ErbB4_Y1188 | ErbB4_ErbB4 | ErbB2_ErbB4 | ErbB3_ErbB4 | EGFR_ErbB4 |
| NIK | Nck | TAK1 |
| ErbB2_Y1023 | ErbB2_ErbB4 | ErbB2_ErbB3 | EGFR_ErbB2 |
| PP2A | (((EGFR_EGFR_TGFa_End) & !(PP2A)) | ((EGFR_EGFR_TGFa_CCP) & !(PP2A)) | ((EGFR_EGFR_TGFa_PM) & !(PP2A)) | ((EGFR_EGFR_EGF_End) & !(PP2A)) | ((EGFR_EGFR_EGF_PM) & !(PP2A)) | ((EGFR_EGFR_EGF_MVB) & !(PP2A)) | ((EGFR_EGFR) & !(PP2A)) | (PP2A & (((!EGFR_EGFR & !EGFR_EGFR_EGF_CCP & !EGFR_EGFR_EGF_CCV & !EGFR_EGFR_EGF_End & !EGFR_EGFR_EGF_MVB & !EGFR_EGFR_EGF_PM & !EGFR_EGFR_TGFa_CCP & !EGFR_EGFR_TGFa_CCV & !EGFR_EGFR_TGFa_End & !EGFR_EGFR_TGFa_PM & !EGFR_ErbB2 & !EGFR_ErbB3 & !EGFR_ErbB4)))) | ((EGFR_EGFR_EGF_CCV) & !(PP2A)) | ((EGFR_EGFR_EGF_CCP) & !(PP2A)) | ((EGFR_EGFR_TGFa_CCV) & !(PP2A)) | ((EGFR_ErbB4) & !(PP2A)) | ((EGFR_ErbB2) & !(PP2A)) | ((EGFR_ErbB3) & !(PP2A))) | !(EGFR_EGFR | EGFR_EGFR_EGF_CCP | EGFR_EGFR_EGF_CCV | EGFR_EGFR_EGF_End | EGFR_EGFR_EGF_MVB | EGFR_EGFR_EGF_PM | EGFR_EGFR_TGFa_CCP | EGFR_EGFR_TGFa_CCV | EGFR_EGFR_TGFa_End | EGFR_EGFR_TGFa_PM | EGFR_ErbB2 | EGFR_ErbB3 | EGFR_ErbB4 | PP2A) |
| IP3 | (PLC_B & PIP2_45) | (PLC_g & PIP2_45) |
| Gbg_s | (alpha_sR & (!Gas & !Gbg_s)) | Gas |
| PKC | ((AA & (Ca & PKC_primed)) & !(Trx & PKC)) | ((DAG & (Ca & PKC_primed)) & !(Trx & PKC)) | ((PKC & (!PP2A & !Trx)) & !(Trx & PKC)) |
| Epsin | EGFR_Ub | EGFR_EGFR_TGFa_PM | EGFR_EGFR_EGF_PM | PIP2_45 |
| PA | PLD |
| RIN | Ras |
| PIP_4 | (((PIP_4 & (!PI3K & !PI5K)) & !(PI5K & PIP_4)) & !(PI3K & PIP_4)) | (((PTEN & (!PIP_4 & PIP2_34)) & !(PI5K & PIP_4)) & !(PI3K & PIP_4)) | (((PI4K & !PIP_4) & !(PI5K & PIP_4)) & !(PI3K & PIP_4)) |
| EGFR_T654 | PKC |
| AP2 | CIN85 | EGFR_EGFR_TGFa_PM |EGFR_EGFR_EGF_PM | PIP3_345 | PIP2_45 | Hip1R | Epsin | Eps15 |
| ESCRT_0 | EGFR_EGFR_EGF_End & PIP3_345 |
| EGFR_ErbB4 | EGF & (!EGFR_T654 & !ErbB2_Free & !ErbB3_Free & NRG & EGFR_Free & ErbB4_Free) |
| Rho | (p115RhoGEF & !Rho & !RhoGDI & p120_catenin) | (Rho & !Graf & !PKA & !p190RhoGAP) |
| Tiam | (CaMK & ((PIP2_34 | PIP3_345) & (PIP2_45 | Rap1 | Ras))) | (Src & ((PIP2_34 | PIP3_345) & (PIP2_45 | Rap1 | Ras))) | (PKC & ((PIP2_34 | PIP3_345) & (PIP2_45 | Rap1 | Ras))) |
| Hakai | Src & !Ca & E_cadherin |
| ErbB2_Lysosome | ErbB2_ErbB3 & ErbB2Deg_Contr | (ErbB2_Ub & !ErbB2Deg_Contr) |
| RhoK | Rho |
| p115RhoGEF | Ga_1213 & PIP3_345 |
| Rabenosyn_5 | Rab5 & PIP3_345 |
| Sos | (Nck & Crk & PIP3_345) | (Crk & (((!Erk & !Nck & !PIP2_45)) & Grb2 & PIP3_345)) | ((Grb2 & PIP3_345) & !Cbl_RTK) |
| Eps15 | EGFR_Ub | EGFR_EGFR_TGFa_PM | EGFR_EGFR_EGF_PM |
| RhoGDI | (!(AA | PIP2_45 | PKC)) | !(AA | PIP2_45 | PKC) |
| Grb2 | (Src & Fak) | Shc | ErbB2_Y1139 | EGFR_Y1086 | EGFR_Y1068 |
| ILK | PIP3_345 |
| ErbB2_Y1139 | ErbB2_ErbB4 | ErbB2_ErbB3 | EGFR_ErbB2 |
| EGFR_EGFR_EGF_CCP | (EGFR_EGFR_EGF_CCP & !EGFR_EGFR_EGF_CCV) | ((AP2 & Cbl_RTK & Clathrin & EGFR_EGFR_EGF_PM & PIP2_45 & Rab5) & !EGFR_EGFR_EGF_CCV) | ((Epsin & (Cbl_RTK & Clathrin & EGFR_EGFR_EGF_PM & PIP2_45 & Rab5)) & !EGFR_EGFR_EGF_CCV) | ((Eps15 & Cbl_RTK & Clathrin & EGFR_EGFR_EGF_PM & PIP2_45 & Rab5) & !EGFR_EGFR_EGF_CCV) |
| alpha_qR | (alpha_qR & !(B_Arrestin & (((!Palpha_iR & !alpha_qL & !alpha_qR)) | Palpha_iR))) | (alpha_qL & !(B_Arrestin & (((!Palpha_iR & !alpha_qL & !alpha_qR)) | Palpha_iR))) | ((Palpha_iR & !B_Arrestin) & !(B_Arrestin & (((!Palpha_iR & !alpha_qL & !alpha_qR)) | Palpha_iR))) |
| Cbl_RTK | ((Grb2 & Src & !(CIN85 & Spry2)) & !EGFR_T654) | ((EGFR_Y1045 & Src & !(CIN85 & Spry2)) & !EGFR_T654) |
| GCK | Trafs |
| Dynamin | EGFR_EGFR_TGFa_PM | EGFR_EGFR_EGF_PM | PIP2_45 | Endophilin | Grb2 |
| ESCRT_II | ESCRT_I |
| PI3K | (Gab1 & !PI3K_I) | (ErbB3_Y127 & !PI3K_I) | (Crk & !PI3K_I) | ((Src & Cbl_RTK) & !PI3K_I | (Gbg_i & !PI3K_I) | (Fak & !PI3K_I) | (ErbB3_Y1035 & !PI3K_I) | (ErbB3_Y1178 & !PI3K_I) | (E_cadherin & !PI3K_I) | (Ras & !PI3K_I) | (ErbB3_Y1203_05 & !PI3K_I) | (EGFR_Y845 & !PI3K_I) | (ErbB3_Y1241 & !PI3K_I) | (ErbB4_Y1056 & !PI3K_I) | (ErbB3_Y1257 & !PI3K_I) | (EGFR_Y920 & !PI3K_I) |
| CaM | Ca |
| Ras | SHP2 | Sos | RasGRF_GRP |
| Palpha_sR | alpha_sR & GRK |
| ESCRT_I | ESCRT_0 |
| ErbB3_Y1180 | ErbB2_ErbB3 | ErbB3_ErbB4 | EGFR_ErbB3 |
| B_Parvin | ILK |
| Raf_Loc | (Ras & (!Raf_Loc & Raf_DeP)) | (Raf_Loc & !Raf) |
| Graf | Fak & Src |
| p38 | ((MKK3 & !PP2A) & !MKPs) | ((MKK6 & !PP2A) & !MKPs) | ((Sek1 & !PP2A) & !MKPs) |
| DOCK180 | Crk & Cas & PIP3_345 |
| Shc | ((ErbB4_Y1242 & (((EGFR_EGFR | EGFR_EGFR_EGF_CCP | EGFR_EGFR_EGF_CCV | EGFR_EGFR_EGF_End | EGFR_EGFR_EGF_MVB | EGFR_EGFR_EGF_PM | EGFR_EGFR_TGFa_CCP | EGFR_EGFR_TGFa_CCV | EGFR_EGFR_TGFa_End | EGFR_EGFR_TGFa_PM | EGFR_ErbB2 | EGFR_ErbB3 | EGFR_ErbB4 | ErbB2_ErbB4 | ErbB3_ErbB4)))) & !PTEN) | ((ErbB2_Y1248 & (((EGFR_EGFR | EGFR_EGFR_EGF_CCP | EGFR_EGFR_EGF_CCV | EGFR_EGFR_EGF_End | EGFR_EGFR_EGF_MVB | EGFR_EGFR_EGF_PM | EGFR_EGFR_TGFa_CCP | EGFR_EGFR_TGFa_CCV | EGFR_EGFR_TGFa_End | EGFR_EGFR_TGFa_PM | EGFR_ErbB2 | EGFR_ErbB3 | EGFR_ErbB4 | ErbB2_ErbB4 | ErbB3_ErbB4)))) & !PTEN) | (Src & !PTEN) | ((ErbB4_Y1188 & (((EGFR_EGFR | EGFR_EGFR_EGF_CCP | EGFR_EGFR_EGF_CCV | EGFR_EGFR_EGF_End | EGFR_EGFR_EGF_MVB | EGFR_EGFR_EGF_PM | EGFR_EGFR_TGFa_CCP | EGFR_EGFR_TGFa_CCV | EGFR_EGFR_TGFa_End | EGFR_EGFR_TGFa_PM | EGFR_ErbB2 | EGFR_ErbB3 | EGFR_ErbB4 | ErbB2_ErbB4 | ErbB3_ErbB4)))) & !PTEN) | ((ErbB2_Y1196 & (((EGFR_EGFR | EGFR_EGFR_EGF_CCP | EGFR_EGFR_EGF_CCV | EGFR_EGFR_EGF_End | EGFR_EGFR_EGF_MVB | EGFR_EGFR_EGF_PM | EGFR_EGFR_TGFa_CCP | EGFR_EGFR_TGFa_CCV | EGFR_EGFR_TGFa_End | EGFR_EGFR_TGFa_PM | EGFR_ErbB2 | EGFR_ErbB3 | EGFR_ErbB4 | ErbB2_ErbB4 | ErbB3_ErbB4)))) & !PTEN) | ((EGFR_Y1173 & (((EGFR_EGFR | EGFR_EGFR_EGF_CCP | EGFR_EGFR_EGF_CCV | EGFR_EGFR_EGF_End | EGFR_EGFR_EGF_MVB | EGFR_EGFR_EGF_PM | EGFR_EGFR_TGFa_CCP | EGFR_EGFR_TGFa_CCV | EGFR_EGFR_TGFa_End | EGFR_EGFR_TGFa_PM | EGFR_ErbB2 | EGFR_ErbB3 | EGFR_ErbB4 | ErbB2_ErbB4 | ErbB3_ErbB4)))) & !PTEN) | (Fak & !PTEN) | ((EGFR_Y1148 & (((EGFR_EGFR | EGFR_EGFR_EGF_CCP | EGFR_EGFR_EGF_CCV | EGFR_EGFR_EGF_End | EGFR_EGFR_EGF_MVB | EGFR_EGFR_EGF_PM | EGFR_EGFR_TGFa_CCP | EGFR_EGFR_TGFa_CCV | EGFR_EGFR_TGFa_End | EGFR_EGFR_TGFa_PM | EGFR_ErbB2 | EGFR_ErbB3 | EGFR_ErbB4 | ErbB2_ErbB4 | ErbB3_ErbB4)))) & !PTEN) | ((ErbB2_Y1221_22 & (((EGFR_EGFR | EGFR_EGFR_EGF_CCP | EGFR_EGFR_EGF_CCV | EGFR_EGFR_EGF_End | EGFR_EGFR_EGF_MVB | EGFR_EGFR_EGF_PM | EGFR_EGFR_TGFa_CCP | EGFR_EGFR_TGFa_CCV | EGFR_EGFR_TGFa_End | EGFR_EGFR_TGFa_PM | EGFR_ErbB2 | EGFR_ErbB3 | EGFR_ErbB4 | ErbB2_ErbB4 | ErbB3_ErbB4)))) & !PTEN) | ((EGFR_Y992 & (((EGFR_EGFR | EGFR_EGFR_EGF_CCP | EGFR_EGFR_EGF_CCV | EGFR_EGFR_EGF_End | EGFR_EGFR_EGF_MVB | EGFR_EGFR_EGF_PM | EGFR_EGFR_TGFa_CCP | EGFR_EGFR_TGFa_CCV | EGFR_EGFR_TGFa_End | EGFR_EGFR_TGFa_PM | EGFR_ErbB2 | EGFR_ErbB3 | EGFR_ErbB4 | ErbB2_ErbB4 | ErbB3_ErbB4)))) & !PTEN) | ((ErbB3_Y1309 & (((EGFR_EGFR | EGFR_EGFR_EGF_CCP | EGFR_EGFR_EGF_CCV | EGFR_EGFR_EGF_End | EGFR_EGFR_EGF_MVB | EGFR_EGFR_EGF_PM | EGFR_EGFR_TGFa_CCP | EGFR_EGFR_TGFa_CCV | EGFR_EGFR_TGFa_End | EGFR_EGFR_TGFa_PM | EGFR_ErbB2 | EGFR_ErbB3 | EGFR_ErbB4 | ErbB2_ErbB4 | ErbB3_ErbB4)))) & !PTEN) |
| Gbg_1213 | alpha_1213R & !Ga_1213 & !Gbg_1213 | Ga_1213 |
| Myosin | ((Myosin & !MLCP) & !(MLCP & Myosin)) | ((RhoK & (!MLCP | !Myosin)) & !(MLCP & Myosin)) | ((PAK & (!MLCP | !Myosin)) & !(MLCP & Myosin)) | ((ILK & (!MLCP | !Myosin)) & !(MLCP & Myosin)) | ((CaM & (!Myosin & MLCK)) & !(MLCP & Myosin)) | ((MLCK & (!MLCP & CaM)) & !(MLCP & Myosin)) |
| ErbB3_Y1203_05 | ErbB2_ErbB3 | ErbB3_ErbB4 | EGFR_ErbB3 |
| p120_catenin | (Rho & !Fer) | EGFR_EGFR_TGFa_End | EGFR_EGFR_TGFa_CCP | EGFR_EGFR_TGFa_PM | EGFR_EGFR_EGF_End | Src | EGFR_EGFR_EGF_PM | EGFR_EGFR_EGF_MVB | EGFR_EGFR | EGFR_EGFR_EGF_CCV | EGFR_EGFR_EGF_CCP | EGFR_EGFR_TGFa_CCV | EGFR_ErbB4 | EGFR_ErbB2 | EGFR_ErbB3 |
| Cortactin | Dynamin | Fer | Src | Rac | PAK | Hip1R | Actin | Erk |
| PTPPEST | Integrins & ECM & !PKA & !PKC |
| EGFR_Y1045 | EGFR_EGFR_TGFa_PM | EGFR_EGFR_EGF_PM | EGFR_ErbB4 | EGFR_ErbB2 | EGFR_ErbB3 | EGFR_EGFR |
| CALM | PIP2_45 |
| PTPa | PKC |
| ErbB2_Free | ((((ErbB2_Free & !ErbB2_ErbB4) & !ErbB2_ErbB3) & !Trastuzumab) & !EGFR_ErbB2) | (ErbB2_Contr & !Trastuzumab) |
| ARNO | PIP2_45 |
| SAPK | ((Sek1 & !(PP2A & SAPK)) & !(MKPs & SAPK)) | ((MKK7 & !(PP2A & SAPK)) & !(MKPs & SAPK)) |
| ErbB3_Y1270 | ErbB2_ErbB3 | ErbB3_ErbB4 | EGFR_ErbB3 |
| EGFR_EGFR_EGF_SR | EGFR_EGFR_EGF_MVB & AMSH |
| EGFR_EGFR_EGF_End | (EGFR_EGFR_EGF_End & !EGFR_EGFR_EGF_MVB) | ((EGFR_EGFR_EGF_CCV & EEA1 & GAK & Hsc70 & PIP3_345 & Rab5 & Rabaptin_5) & !EGFR_EGFR_EGF_MVB) |
| Rab5 | (RIN & !(Rab7 & Rab5)) | (EGFR_EGFR_TGFa_PM & !(Rab7 & Rab5)) | (p120RasGAP & !(Rab7 & Rab5)) | (EGFR_EGFR_EGF_PM & !(Rab7 & Rab5)) | (Rabex_5 & !(Rab7 & Rab5)) | (Rab5 & !(Rab7 & Rab5)) |
| ESCRT_III | ESCRT_II | ESCRT_I |
| PIP3_345 | (PI5K & PIP2_34 & !(PTEN & PIP3_345)) | (PI3K & PIP2_45 & !(PTEN & PIP3_345)) |
| MKK6 | (Tpl2 & ASK1) | (Mekk4 & ASK1) | (PAK & ASK1) | (TAO_12 & ASK1) | (MLK3 & ASK1) | (TAK1 & ASK1) |
| Csk | (Cbp & ((!Gbg_1213 & !Gbg_i & !Gbg_q & !PKA & !SHP2) | (Gbg_1213 | Gbg_i | Gbg_q | PKA))) | ((Fak & (Cbp & Src) & !SHP2) |
| Spry2 | ((EGFR_EGFR_TGFa_End) & !(Cbl_RTK)) | ((EGFR_EGFR_TGFa_CCP) & !(Cbl_RTK)) | ((EGFR_EGFR_TGFa_PM) & !(Cbl_RTK)) | ((EGFR_EGFR_EGF_End) & !Cbl_RTK) | ((EGFR_EGFR_EGF_PM) & !Cbl_RTK) | (EGFR_EGFR_EGF_MVB & !Cbl_RTK) | (EGFR_EGFR & !Cbl_RTK) | (EGFR_EGFR_EGF_CCV & !Cbl_RTK) | (EGFR_EGFR_EGF_CCP & !Cbl_RTK) | (EGFR_EGFR_TGFa_CCV & !Cbl_RTK) | (EGFR_ErbB4 & !Cbl_RTK) | (EGFR_ErbB2 & !Cbl_RTK) | (EGFR_ErbB3 & !Cbl_RTK) |
| GRK | (((B_Arrestin & Src) & !RKIP) & !Erk) | (((Gbg_q & PIP2_45) & !RKIP) & !Erk) | (((Gbg_1213 & PIP2_45) & !RKIP) & !Erk) | (((Gbg_i & PIP2_45) & !RKIP) & !Erk) | (((Gbg_s & PIP2_45) & !RKIP) & !Erk) |
| ErbB3_Y1257 | ErbB2_ErbB3 | ErbB3_ErbB4 | EGFR_ErbB3 |
| EGFR_Y891 | Src |
| ErbB2_Y1248 | ErbB2_ErbB4 | ErbB2_ErbB3 | EGFR_ErbB2 |
| Nck | EGFR_EGFR_TGFa_End | EGFR_EGFR_TGFa_CCP | EGFR_EGFR_TGFa_PM | EGFR_EGFR_EGF_End | EGFR_EGFR_EGF_PM | EGFR_EGFR_EGF_MVB | EGFR_EGFR | Cas | EGFR_EGFR_EGF_CCV | EGFR_EGFR_EGF_CCP | EGFR_EGFR_TGFa_CCV | EGFR_ErbB4 | EGFR_ErbB2 | EGFR_ErbB3 |
| ErbB4_Y1242 | ErbB4_ErbB4 | ErbB2_ErbB4 | ErbB3_ErbB4 | EGFR_ErbB4 |
| Raf | (Ras & Raf) | (Raf & (!Akt & !Erk & !PKA)) | (Src & (!Raf & (PAK & RKIP & Raf_Loc))) | (PAK & ((!Akt & !Erk & !Ras) & Raf)) |
| DAG | (DAG & !DGK) | ((PLC_B & PIP2_45) & !(DGK & DAG)) | ((PLC_g & PIP2_45) & !(DGK & DAG)) |
| Hsp90 | ((ErbB2_ErbB4) & !(CHIP & Hsp90)) | ((ErbB2_ErbB3) & !(CHIP & Hsp90)) | (EGFR_ErbB2 & !(CHIP & Hsp90)) |
| Fer | E_cadherin & p120_catenin |
| Tpl2 | Trafs |
| EGFR_T669 | Erk |
| MKK3 | (Tpl2 & ASK1) | (Mekk4 & ASK1) | (Mekk3 & ASK1) | (Mekk2 & ASK1) | (PAK & ASK1) | (TAO_12 & ASK1) | (MLK1 & ASK1) | (MLK2 & ASK1) | (MLK3 & ASK1) | (TAK1 & ASK1) |
| Actin | (Arp_23 & !IQGAP1 & Myosin & alpha_catenin) | (IQGAP1 & Myosin) |
| Tab_12 | Trafs & !p38 |
| PI5K | (PI5K & Talin) | RhoK | ARF | (Src & (!PI5K & !Talin & Fak)) | PA |
| IL1_TNFR | IL1_TNF |
| Integrins | (Talin & !ILK & !Integrins & ECM) | (PP2A & !Integrins & ECM & ILK & Talin) | (Integrins & !ILK & !Src) | (Src & !ECM & !ILK & !Integrins & !PP2A & !Talin) |
| PIP2_34 | (PIP2_34 & !PI5K & !PTEN) | (PI4K & !PIP2_34 & PI3K) |
| PKC_primed | (PDK1 & !PKC) | (PKC & !PKC_primed & PDK1) | (PKC_primed & !PKC) |
| Ga_1213 | (alpha_1213R & !Ga_1213 & !Gbg_1213) | (Ga_1213 & (!p115RhoGEF & Gbg_1213)) |
| E_cadherin | (((B_catenin & ExtE_cadherin) & !(Hakai & !p120_catenin)) & !(IQGAP1 & !Cdc42 & !Rac)) |
| ErbB2_ErbB4 | NRG & ErbB2_Free & ErbB4_Free & !ErbB3_Free |
| CHIP | AG & Hsp90 |
| Cbp | Src & !SHP2 |
| Raf_Rest | ((Raf_DeP & !Raf & !Raf_Rest) | (Raf_Rest & !Raf_DeP)) | !(Raf | Raf_DeP | Raf_Rest) |
| ErbB3_Free | ErbB3_Contr | (((ErbB3_Free & !ErbB2_ErbB3) & !ErbB3_ErbB4) & !EGFR_ErbB3) |
| Rabex_5 | Rabaptin_5 |
| PLD | (Rho & (!ARF & ((Actin) & (PIP2_45 | PIP3_345)))) | (ARF & (PIP2_45 | PIP3_345)) | (Rac & (!ARF & (Actin & (PIP2_45 | PIP3_345)))) | (Cdc42 & (!ARF & (Actin & (PIP2_45 | PIP3_345)))) | (PKC & (!ARF & ((Actin) & (PIP2_45 | PIP3_345)))) |
| PLA2 | (CaMK & Ca) | (PIP3_345 & (CaMK & PIP2_45)) | (PIP2_45 & (Erk & PIP3_345)) | (Erk & Ca) |
| EGFR_EGFR | (alpha_qR & (Ca & EGFR_Free & EGFR_T654)) | (alpha_1213R & (Ca & EGFR_Free & EGFR_T654)) | (alpha_iR & (Ca & EGFR_Free & EGFR_T654)) |
| Akt | (PDK1 & (!Akt & (PIP2_34 | PIP3_345) & (ILK & Src))) | (CaMKK & (!Akt & (PIP2_34 | PIP3_345) & (ILK & Src))) | (Akt & !PP2A) |
| Trx | Stress | Trafs |
| RGS | CaM & PIP3_345 |
| alpha_iR | (alpha_iR & !(B_Arrestin & (!Palpha_iR & !alpha_iL & !alpha_iR) | Palpha_iR)) | (alpha_iL & !(B_Arrestin & ((!Palpha_iR & !alpha_iL & !alpha_iR) | Palpha_iR))) | ((Palpha_iR & !B_Arrestin) & !(B_Arrestin & (!Palpha_iR & !alpha_iL & !alpha_iR) | Palpha_iR)) |
| CIN85 | Cbl_RTK |
| EGFR_Y1068 | EGFR_EGFR_TGFa_PM| EGFR_EGFR_EGF_PM | EGFR_ErbB4 | EGFR_ErbB2 | EGFR_ErbB3 | EGFR_EGFR |
| ErbB2_Y1221_22 | ErbB2_ErbB4 | ErbB2_ErbB3 | EGFR_ErbB2 |
| PKA | (PDK1 & cAMP & !(PP2A & PKA)) | ((PKA & cAMP) & !(PP2A & PKA)) |
| ErbB2_Ub | (Cbl_ErbB2 & !Hsp90) | CHIP |
| EGFR_Y1101 | Sr |
| EGFR_Ub | (((EGFR_EGFR_TGFa_PM & Cbl_RTK) & !EGFR_EGFR_TGFa_CCP) & !EGFR_EGFR_EGF_CCP) | (((EGFR_EGFR_EGF_PM & Cbl_RTK) & !EGFR_EGFR_TGFa_CCP) & !EGFR_EGFR_EGF_CCP) |
| Arp_23 | WASP |
| RKIP | PKC |
| Cbl_FA | Src & Cdc42 & Pix_Cool & !Cbl_RTK |
| EGFR_EGFR_TGFa_End | ((EGFR_EGFR_TGFa_End) & !EGFR_Free) | (EGFR_EGFR_TGFa_CCV & (((EEA1 & Hsc70 & PIP3_345 & Rab5) & (GAK | Rabaptin_5)))) |
| Palpha_iR | (alpha_iR & GRK) |
| Mekk2 | ((Src & (((EGFR_EGFR_EGF_CCP | EGFR_EGFR_EGF_CCV | EGFR_EGFR_EGF_End | EGFR_EGFR_EGF_MVB | EGFR_EGFR_EGF_PM | EGFR_EGFR_TGFa_CCP | EGFR_EGFR_TGFa_CCV | EGFR_EGFR_TGFa_End | EGFR_EGFR_TGFa_PM | EGFR_ErbB2 | EGFR_ErbB3 | EGFR_ErbB4)))) & !Mekk2) | ((PI3K & (((EGFR_EGFR_EGF_CCP | EGFR_EGFR_EGF_CCV | EGFR_EGFR_EGF_End | EGFR_EGFR_EGF_MVB | EGFR_EGFR_EGF_PM | EGFR_EGFR_TGFa_CCP | EGFR_EGFR_TGFa_CCV | EGFR_EGFR_TGFa_End | EGFR_EGFR_TGFa_PM | EGFR_ErbB2 | EGFR_ErbB3 | EGFR_ErbB4)))) & !Mekk2) | ((Grb2 & (((EGFR_EGFR_EGF_CCP | EGFR_EGFR_EGF_CCV | EGFR_EGFR_EGF_End | EGFR_EGFR_EGF_MVB | EGFR_EGFR_EGF_PM | EGFR_EGFR_TGFa_CCP | EGFR_EGFR_TGFa_CCV | EGFR_EGFR_TGFa_End | EGFR_EGFR_TGFa_PM | EGFR_ErbB2 | EGFR_ErbB3 | EGFR_ErbB4)))) & !Mekk2) | ((PLC_g & (((EGFR_EGFR_EGF_CCP | EGFR_EGFR_EGF_CCV | EGFR_EGFR_EGF_End | EGFR_EGFR_EGF_MVB | EGFR_EGFR_EGF_PM | EGFR_EGFR_TGFa_CCP | EGFR_EGFR_TGFa_CCV | EGFR_EGFR_TGFa_End | EGFR_EGFR_TGFa_PM | EGFR_ErbB2 | EGFR_ErbB3 | EGFR_ErbB4)))) & !Mekk2) |
| IP3R1 | (((PKA & !(PP2A & IP3R1)) & !(CaM & (Ca & IP3R1))) & !(Ca & (!IP3 & IP3R1))) | ((IP3 & Ca & !(CaM & (Ca & IP3R1))) & !(Ca & (!IP3 & IP3R1))) | (((Gbg_i & !(CaM & (Ca & IP3R1))) & !(Ca & (!IP3 & IP3R1))) & !(IP3R1 & (((!Ca & !IP3 & !PKA & !PP2A)) & CaM & Gbg_i))) |
| Palpha_1213R | alpha_1213R & GRK |
| ErbB3_Y1309 | ErbB2_ErbB3 | ErbB3_ErbB4 | EGFR_ErbB3 |
| VPS4 | ESCRT_III |
| IQGAP1 | (!(CaM & Ca)) | !(Ca | CaM) |
| Hsc70 | Dynamin | GAK |
| Gai | (alpha_iR & !Gai & !Gbg_i) | (PKA & (!Gai & !Gbg_i & !alpha_sR & alpha_sL)) | (Gbg_i & (!RGS & Gai)) |
| Ca | IP3R1 & ExtPump |
| Palpha_qR | alpha_qR & GRK |
| ErbB3_ErbB4 | NRG & ErbB3_Free & ErbB4_Free & !ErbB2_Free |
| WASP | ((Src & (((Grb2 | Nck | PIP2_45)) & Cdc42 & Crk)) & !PTPPEST) | ((Cdc42 & ((!Crk & !PTPPEST) & (Grb2 | Nck | PIP2_45) & (Fak | Src))) & !PTPPEST) | ((Fak & ((Grb2 | Nck | PIP2_45) & Cdc42 & Crk)) & !PTPPEST) |
| Gbg_q | Gaq | alpha_qR & !Gaq & !Gbg_q |
| PDE4 | (B_Arrestin & !Erk) | (PKA & B_Arrestin) |
| MLCP | (PKA & RhoK& !Raf & !PAK & !ILK & !PKC) | !(ILK | PAK | PKA | PKC | Raf | RhoK) |
| ErbB2_Y1196 | (ErbB2_ErbB4) | (ErbB2_ErbB3) | (EGFR_ErbB2) |
| Ral | RalGDS | CaM | AND_34 |
| Cas | (Src & Fak & !(PTPPEST & Cas)) |
| B_catenin | ((PTP1b & (((!EGFR_EGFR & !EGFR_EGFR_EGF_CCP & !EGFR_EGFR_EGF_CCV & !EGFR_EGFR_EGF_End & !EGFR_EGFR_EGF_MVB & !EGFR_EGFR_EGF_PM & !EGFR_EGFR_TGFa_CCP & !EGFR_EGFR_TGFa_CCV & !EGFR_EGFR_TGFa_End & !EGFR_EGFR_TGFa_PM & !EGFR_ErbB2 & !EGFR_ErbB3 & !EGFR_ErbB4 & !Src)))) | (Fer & (((!EGFR_EGFR & !EGFR_EGFR_EGF_CCP & !EGFR_EGFR_EGF_CCV & !EGFR_EGFR_EGF_End & !EGFR_EGFR_EGF_MVB & !EGFR_EGFR_EGF_PM & !EGFR_EGFR_TGFa_CCP & !EGFR_EGFR_TGFa_CCV & !EGFR_EGFR_TGFa_End & !EGFR_EGFR_TGFa_PM & !EGFR_ErbB2 & !EGFR_ErbB3 & !EGFR_ErbB4 & !Src)) | PTP1b))) | !(EGFR_EGFR | EGFR_EGFR_EGF_CCP | EGFR_EGFR_EGF_CCV | EGFR_EGFR_EGF_End | EGFR_EGFR_EGF_MVB | EGFR_EGFR_EGF_PM | EGFR_EGFR_TGFa_CCP | EGFR_EGFR_TGFa_CCV | EGFR_EGFR_TGFa_End | EGFR_EGFR_TGFa_PM | EGFR_ErbB2 | EGFR_ErbB3 | EGFR_ErbB4 | Fer | PTP1b | Src) |
| Sek1 | (Tpl2 & ASK1) | (Mekk4 & ASK1) | (Mekk3 & ASK1) | (Mekk2 & ASK1) | (Mekk1 & ASK1) | (MLK1 & ASK1) | (MLK2 & ASK1) | (MLK3 & ASK1) | (TAK1 & ASK1) |
| Trafs | IL1_TNFR |
| Mekk3 | (IL1_TNFR & !Gab1) | (Rac & !Gab1) | (Trafs & !Gab1) |
| EGFR_EGFR_TGFa_CCP | (EGFR_EGFR_TGFa_CCP & !EGFR_EGFR_TGFa_CCV) | (AP2 & (EGFR_EGFR_TGFa_PM & Rab5 & Clathrin & PIP2_45)) | (Epsin & (Rab5 & EGFR_EGFR_TGFa_PM & Clathrin & PIP2_45)) | (Eps15 & Rab5 & EGFR_EGFR_TGFa_PM & Clathrin & PIP2_45) |
| alpha_sR | ((Palpha_sR & !B_Arrestin) & !(B_Arrestin & (((!Palpha_sR & !alpha_sL & !alpha_sR)) | Palpha_sR))) | (alpha_sR & !(B_Arrestin & ((!Palpha_sR & !alpha_sL & !alpha_sR) | Palpha_sR))) | (alpha_sL & !(B_Arrestin & ((!Palpha_sR & !alpha_sL & !alpha_sR) | Palpha_sR))) |
| PTP1b | (!(EGFR_EGFR_TGFa_CCP | EGFR_EGFR_TGFa_PM | EGFR_EGFR_EGF_End | EGFR_EGFR_EGF_PM | EGFR_EGFR_EGF_MVB | Stress | EGFR_EGFR | EGFR_EGFR_EGF_CCV | EGFR_EGFR_EGF_CCP | EGFR_EGFR_TGFa_CCV | EGFR_ErbB4 | EGFR_ErbB2 | EGFR_ErbB3)) | !(EGFR_EGFR | EGFR_EGFR_EGF_CCP | EGFR_EGFR_EGF_CCV | EGFR_EGFR_EGF_End | EGFR_EGFR_EGF_MVB | EGFR_EGFR_EGF_PM | EGFR_EGFR_TGFa_CCP | EGFR_EGFR_TGFa_CCV | EGFR_EGFR_TGFa_PM | EGFR_ErbB2 | EGFR_ErbB3 | EGFR_ErbB4 | Stress) |
| EGFR_Y845 | (EGFR_Free & Cas & Integrins & Src) | (Src & EGFR_EGFR_EGF_PM) |
| EEA1 | Rab5 & PIP3_345 |
| AMSH | Alix | ESCRT_0 | ESCRT_I |
| GAK | (EGFR_EGFR_TGFa_CCP & (AP2 | Clathrin | Dynamin)) | (EGFR_EGFR_EGF_CCP & (AP2 | Clathrin | Dynamin)) | (GAK & (EGFR_EGFR_EGF_CCV | EGFR_EGFR_TGFa_CCV)) |
| EGFR_EGFR_TGFa_PM | (((EGFR_Free & (!EGFR_T654 & TGFa)) & !EGFR_EGFR_TGFa_CCP) & !ErbB2_Free) | (EGFR_EGFR_TGFa_PM & !EGFR_EGFR_TGFa_CCP) |
| Rab7 | Rab5 |
| DGK | EGFR_EGFR_TGFa_End | EGFR_EGFR_TGFa_CCP | EGFR_EGFR_TGFa_PM | EGFR_EGFR_EGF_End | (Src & Ca & PA) | EGFR_EGFR_EGF_PM | EGFR_EGFR_EGF_MVB | (PKC & DAG) | EGFR_EGFR | EGFR_EGFR_EGF_CCV | EGFR_EGFR_EGF_CCP | EGFR_EGFR_TGFa_CCV | EGFR_ErbB4 | EGFR_ErbB2 | EGFR_ErbB3 |
| PTEN | (Stress & !PTEN_I) | ((Pix_Cool & Cdc42 & PI3K & Rho) & !PTEN_I) |
